# Computational Network Modeling of Intranidal Hemodynamic Compartmentalization in a Theoretical Three-Dimensional Brain Arteriovenous Malformation

**DOI:** 10.3389/fphys.2019.01250

**Published:** 2019-09-24

**Authors:** Mika S. Jain, Huy M. Do, Tarik F. Massoud

**Affiliations:** ^1^Department of Physics, School of Humanities and Sciences, Stanford University, Stanford, CA, United States; ^2^Department of Computer Science, School of Engineering, Stanford University, Stanford, CA, United States; ^3^Division of Neuroimaging and Neurointervention, Department of Radiology, School of Medicine, Stanford University, Stanford, CA, United States; ^4^Department of Neurosurgery, School of Medicine, Stanford University, Stanford, CA, United States

**Keywords:** angiography, embolization, nidus, plexiform, theoretical

## Abstract

There are currently no *in vivo* techniques to accurately study dynamic equilibrium of blood flow within separate regions (compartments) of a large brain arteriovenous malformation (AVM) nidus. A greater understanding of this AVM compartmentalization, even if theoretical, would be useful for optimal planning of endovascular and multimodal AVM therapies. We aimed to develop a biomathematical AVM model for theoretical investigations of intranidal regions of increased mean intravascular pressure (P_mean_) and flow representing hemodynamic compartments, upon simulated AVM superselective angiography (SSA). We constructed an AVM model as a theoretical electrical circuit containing four arterial feeders (AF1–AF4) and a three-dimensional nidus of 97 interconnected plexiform and fistulous components. We simulated SSA by increases in P_mean_ in each AF (with and without occlusion of all other AFs), and then used network analysis to establish resulting increases in P_mean_ and flow within each nidus vessel. We analyzed shifts in hemodynamic compartments consequent to increasing AF injection pressures. SSA simulated by increases of 10 mm Hg in AF1, AF2, AF3, or AF4 resulted in dissipation of P_mean_ over 38, 66, 76, or 20% of the nidus, respectively, rising slightly with simultaneous occlusion of other AFs. We qualitatively analyzed shifting intranidal compartments consequent to varying injection pressures by mapping the hemodynamic changes onto the nidus network. Differences in extent of nidus filling upon SSA injections provide theoretical evidence that hemodynamic and angioarchitectural features help establish AVM nidus compartmentalization. This model based on a theoretical AVM will serve as a useful computational tool for further investigations of AVM embolotherapy strategies.

## Introduction

A brain arteriovenous malformation (AVM) is a congenital abnormal tangle (nidus) of dilated blood vessels in the brain that directly diverts blood from arteries (or arterial feeders [AFs]) to veins (or draining veins [DVs]), and in doing so, bypasses normal brain tissue. The two hallmarks of AVMs are the presence of a nidus and the associated hemodynamic shunting of arterial blood at high pressure through the nidus and directly into DVs, without the resistance normally offered by brain capillaries. In the absence of head trauma, AVMs are the most common cause of brain hemorrhage in young adults.

Within any large AVM nidus, a dynamic equilibrium of blood flow exists in separate regions, or compartments, that are each supplied by their own AF (Massoud and Hademenos, 1999; Kakizawa et al., 2002). The precise hemodynamic factors that determine the extent and nature of nidus compartments have long been debated but not investigated in any detail. A greater theoretical understanding of the nature of AVM hemodynamic compartmentalization would be useful for optimal planning of endovascular and multimodal AVM treatments.

Importantly, there are currently no practical *in vivo* techniques to accurately study hemodynamic compartmentalization within an AVM nidus. No current imaging or recording technique can provide precise hemodynamic information from within the nidus of a brain AVM. Owing to inaccessibility or danger of direct transvascular access of fragile intranidal microvessels, such an assessment is often reduced to measuring blood flow and/or pressure outside the margins of a nidus using microcatheters placed in AFs and DVs during superselective angiography (SSA). Current imaging techniques are also limited by insufficient spatial resolution to allow visualization of individual intranidal microvessels when attempting to interrogate their flow non-invasively.

Biomathematical models offer an alternative theoretical approach to analyzing AVM hemodynamics, which may be otherwise difficult to quantify especially within or in close proximity to the nidus. Typically, this has been accomplished in the past using simulated elementary feeding and draining pedicle anatomy and a nidus composed of simple single or multiple array(s) of parallel vessels (Lo, 1993; Gao et al., 1998; White and Smith, 2013; Kiran Kumar et al., 2017).

For a brief review of SSA and the current knowledge of intranidal hemodynamic compartmentalization, see Supplementary Material. To more accurately characterize intranidal redistribution of hemodynamic forces following SSA (prior to AVM embolotherapy), a model with a more complex nidus angioarchitecture and more consistent with that of human AVMs must be employed. Hademenos et al. (1996) previously introduced the concept of using a theoretical electrical network to simulate hemodynamics through a complex mesh of interconnected AVM nidus vessels, in the form of a rudimentary two-dimensional (2-D) structure fed and drained by multiple AFs and DVs. Their 2-D computational AVM model was used previously as a theoretical simulator for investigating AVM venous drainage impairment (Hademenos and Massoud, 1996b), AVM radiosurgery (Massoud et al., 2000), and in simulating a newly proposed embolization technique for brain AVMs (Massoud and Hademenos, 1999).

However, owing to practical constraints imposed by complex biomathematical simulations, all previously reported theoretical AVM models have been in essence extremely rudimentary replicas of true brain AVMs, each containing an AVM nidus composed of only a small number of vessels. This radical oversimplification of AVMs makes it impossible to study hemodynamic compartmentalization within a nidus. The electrical network AVM model we present herein attempts to elucidate qualitative hemodynamics within a theoretical nidus by adopting more representative gross morphological, histological, angiographic, and biomechanical characteristics of brain AVMs than available previously.

This more complex model is necessary to address the question of AVM compartmentalization. Compartments are regions of the network each consisting of numerous interconnected vessels. Earlier simple AVM models contained just a few vessels, and therefore have no analogy to intranidal compartments, and there is no way to address the question of hemodynamic compartmentalization. A head-to-head comparison of our model to other earlier models is challenging because most of these prior publications do not release their code or include enough information to rebuild their model, and, most importantly, it is not possible to investigate intranidal compartmentalization with these simpler models because they contain too few vessels. Our goal was not simply to propose a novel and improved model, but also to capitalize on availability of this new, angioarchitecturally more complex and realistic nidus simulator to theoretically investigate an important morphological and hemodynamic feature of AVMs, i.e., nidus compartmentalization.

To that end, we present an electrical network model of a theoretical brain AVM constructed with multiple AFs and DVs, and a three-dimensional (3-D) spatially oriented nidus containing a large number (97) of interconnected plexiform and fistulous intranidal vessels. The inclusion of a simulated intranidal fistula is important because fistulas are of larger caliber than plexiform vessels, and therefore carry higher hemodynamic loads than their plexiform counterparts (Yasargil, 1987b). We use this model to investigate theoretical AVM intranidal hemodynamic compartmentalization after simulated SSA through AFs, as well as the effects of nidus angioarchitecture on spread of simulated contrast medium when injected through AFs. We characterize the hemodynamic conduct of this model as a prelude to its future applications in theoretical simulations of embolotherapy, and the study of potential hemodynamic consequences on the nidus and brain tissue surrounding a treated AVM.

## Materials and Methods

### AVM Hemodynamics

Blood flow within most human vasculature consists of non-Newtonian fluid flowing in a pulsatile nature through viscoelastic, tapered tubes. This requires computationally intensive calculations to simulate with high fidelity. However, since blood flow in AVMs and surrounding vasculature is within thin capillary vessels downstream of high capacitance vessels, turbulence, pulsatility, and changes in viscosity are negligible. AVM hemodynamics can therefore be described sufficiently by the Hagen–Poiseuille equation,

(1) Q=πΔPr/48Lη,

where Q is vessel flow rate, ΔP is the difference in pressure between the ends of the vessel, *r* is vessel inner radius, L is vessel length, and η is the blood viscosity (η = 3.5 centiPoise). This is analogous to Ohm’s law, *I* = *V*/*R*_v_, with current *I* = Q, voltage *V* = ΔP, and vessel resistance *R*_v_ = 8 L η/π *r*^4^. Despite known limitations in the applicability of the Hagen–Poiseuille equation (the fluid must be incompressible and Newtonian, the flow laminar through a pipe of constant circular cross-section that is substantially longer than its diameter, and there is no acceleration of fluid in the pipe), this analogy allows simulation of the fluidic network of blood flow within AVM vasculature using established, matrix-based analysis of electrical networks. If pressure, length, and radius are known for all blood vessels, it is possible to calculate blood flow rate throughout the entire AVM. Importantly, we ignored pressure losses at vessel junctions in our analysis. This is a significant assumption, but since we consider intranidal vessels to be thin, the length of the region at each junction that cannot be approximated as a cylinder is tiny. The pressure drop across a junction may therefore be negligible, and although it ideally should be taken into account, we reasonably did not consider this to limit further complexity of our calculations.

### AVM Network Model

**FIGURE 1:**
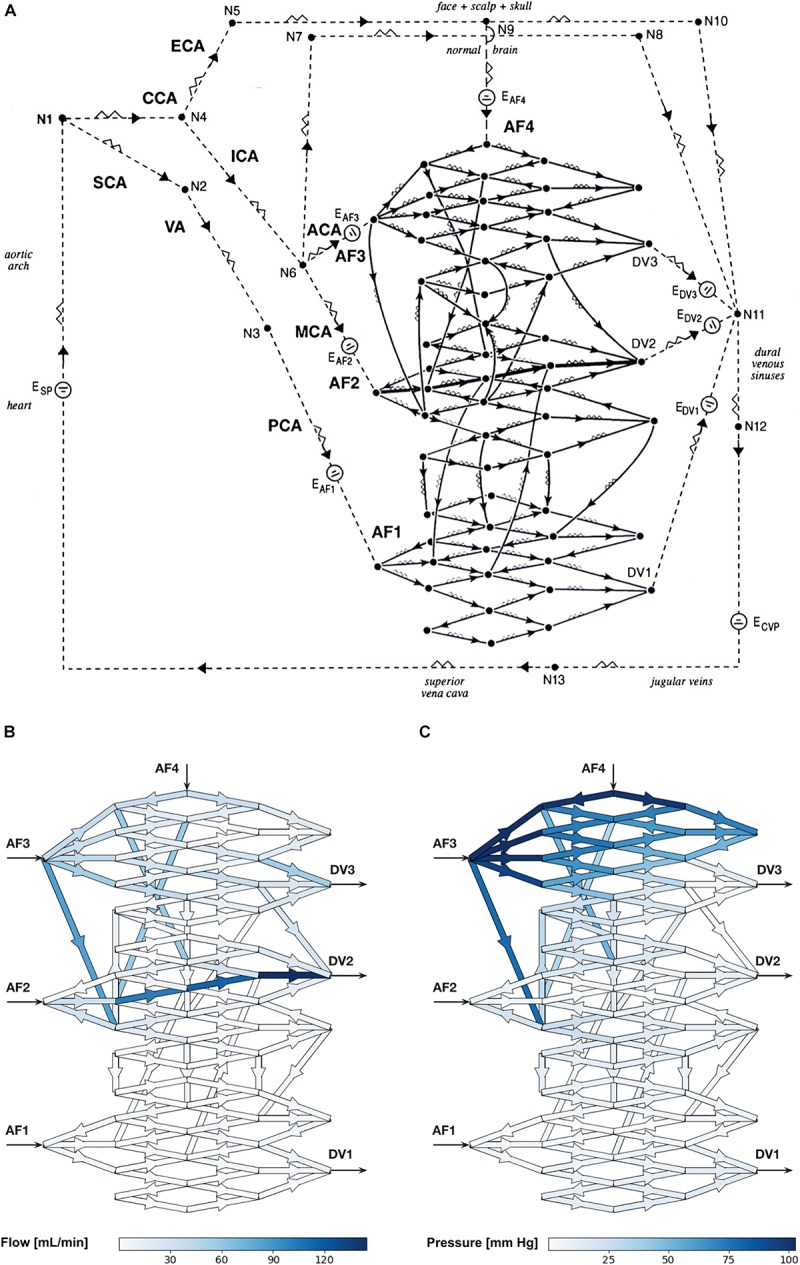
**(A)** Schematic diagram of the electrical circuit showing the biomathematical AVM model and the details of the 3-D AVM nidus network. AF, arterial feeder; DV, draining vein; CCA, common carotid artery; ECA, external carotid artery; ICA, internal carotid artery; SCA, subclavian artery; VA, vertebral artery; PCA, posterior cerebral artery; ACA, anterior cerebral artery; MCA, middle cerebral artery; E, electromotive force; N, node; CVP, central venous pressure. The intranidal fistula spans AF2 to DV2. Arrowheads indicate direction of flow. **(B,C)** Hemodynamic simulations using the biomathematical 3-D AVM model in its baseline state. **(B)** Simulated intranidal volumetric flow rate through the nidus (color scale in mL/min). **(C)** Simulated intranidal P_mean_ through the nidus (color scale in mm Hg). AF, arterial feeder; DV, draining vein.

We constructed an electrical network to closely characterize an AVM situated within the intracranial circulation, and supplied by the three brain vascular territories. Our network consisted of four arterial feeders (AF1–AF4), three draining veins (DV1–DV3), and an arbitrary, randomly chosen nidus angioarchitecture consisting of 97 vessels (93 plexiform, and four fistulous) interconnected in a 3-D morphology, as shown in Figure 1A. Two AFs (AF1 and AF2) were considered major while the other two (AF3 and AF4) were minor. We assigned realistic biophysical values to all model vessels (see Supplementary Table 1). We obtained the average diameter values of the arteries and veins comprising the circulatory network from biomedical literature while the length of each vessel was approximated based on knowledge of gross anatomy. Mean intravascular pressure (P_mean_) values at major points within the extranidal circulatory network were assigned as the arithmetic mean of values published in clinical accounts: mean systemic blood pressure, 74 mm Hg; mean major AF pressure, 47 mm Hg; mean minor AF pressure, 50 mm Hg; mean DV pressure, 17 mm Hg; and mean central venous pressure (CVP), 5 mm Hg. The nidus vessels comprising the plexiform component were held fixed at a radius of 0.05 cm, and the length of each vessel included the incorporation of an arbitrary factor for tortuosity. The intranidal fistula consisted of a direct connection between AF2 and DV2 with branching plexiform vessels, and was maintained at a uniform radius of 0.10 cm. Since there are no values in the literature for the length of the plexiform vessels, they were approximated to be uniform in length (5.0 cm). The fistulous vessel was given a slightly shorter length (4.0 cm) (see Supplementary Table 1).

This model was a more complex and realistic elaboration on a previously published model by Hademenos et al. (1996), which lacked sufficient nidus size and morphological complexity to allow a rigorous analysis of the separate hemodynamic compartments within an AVM nidus intended in this study. Thus, we introduced two main improvements. First, we increased the number of intranidal vessels from 28 to 97, which is many more vessels than any previously published models. The main advantage of this is that it allowed the performance of higher-resolution investigations and simulations of intranidal compartments within anatomically more faithful representations of AVMs. Second, we introduced a model with a 3-D instead of 2-D angioarchitecture. Previous studies have all used AVM networks that were simple planar renderings. To simulate a basic 3-D spatial configuration, our AVM network included vessels that connected to more distant nodes. This departure was necessary to ensure a degree of complexity in a network model that reflects the true angioarchitecture of human AVMs.

### Hemodynamic Simulations

In network analysis, vessels of various radii and lengths are distributed randomly in a dependent manner to resemble a highly disordered frame or network through which fluid will flow. In analogies found in electricity, the circulatory network can be represented by a complex electrical circuit of connected wires with variable resistance through which the current or flow, powered by an electrical power source or pressure gradient, will traverse. Each wire or vessel connection represents a node or a location at which flow converges and diverges. With respect to the AVM, the node resembles the start or end of a vascular branch, e.g., a bifurcation or trifurcation, within the vascular bed.

The flow through the AVM network was calculated according to Kirchhoff’s circuit laws: (1) the algebraic sum of the currents at any node must be zero, i.e., flow into a node is equal to flow out of the node; and (2) the algebraic sum of the changes in potential (pressure gradient) encountered in a closed traversal must be zero. Within a current loop, if a resistor is traversed in the direction of the current, the change in pressure is −QR_v_; in the opposite direction it is +QR_v_. If a source of pressure is traversed in the direction of the pressure increase, the change in pressure is +ΔP and in the opposite direction it is −ΔP. This yields a system of linear equations relating flow, pressure, and resistance. These equations were represented in matrix form,

(2) Δ⁢Δ⁢P=R⁢Qv,

where ΔΔP is a column vector that contains zeros for each instance of Kirchhoff’s first law and pressure changes for each instance of Kirchhoff’s second law. These pressure changes are those accumulated over each closed vessel loop in the network. Because the loops are closed, these pressure changes are mostly zero and only non-zero when there is an external source of pressure difference in the loop, such as at the heart. Q is a column vector of all of the vessel flow rates. *R*_v_ is a matrix with elements that are either various vessel resistances or the values ±1. *R*_v_ is constructed so that each line of matrix Eq. (2) is an instance of either Kirchhoff’s first law or Kirchhoff’s second law. The flow rates through each vessel were then solved by calculating:

(3) $$Q=R_{v}{}^{-1}\Delta \Delta P,$$

where *R*_v_^–1^ is the left inverse of *R*_v_. Calculating *R*_v_^–1^ was a computational bottleneck of our approach, thus limiting the size of our network model to 97 vessels only (which was a random number we chose nonetheless to be substantially higher than in previous AVM models). Calculating Q subsequently allowed us to calculate ΔP for each vessel by solving the Hagen–Poiseuille equation. We performed all calculations and simulation steps using Python^TM^ (Delaware, United States).

We then simulated SSA of the AVM nidus by increasing the intravascular pressure (thus, simulating a contrast medium injection pressure) by 10, or 20, or 30 mm Hg separately within each of the four AFs supplying the AVM. These elevations in pressure owing to the injections are typical of those measured in small vessels of experimental animals when using microcatheters (Saitoh et al., 1996). Downstream intranidal vessels revealing consequent increased P_mean_ and flow during SSA were considered to represent vessels in which contrast medium had spread, as experimentally shown by Saitoh et al. (1996). Moreover, intranidal compartments (also see Supplementary Discussion Section 1.2) were defined by the presence and extent of spread of contrast medium within the nidus, as clinically visualized and recorded on patient angiograms by Kakizawa et al. (2002) after performing SSA, and as discussed by Yasargil (1987a) previously. To study the contribution of each AF to the downstream formation of separate intranidal compartments, without the confounding influence of other AFs, we repeated these simulated injections for each AF in turn while simultaneously occluding all other AFs. We used network analysis to establish consequent values in P_mean_ and flow within each nidus vessel upon SSA.

Therefore, having first conducted hemodynamic simulations with unperturbed AF pressures to determine the baseline P_mean_ and flow within each nidus vessel prior to SSA injections, we then used these baseline simulations to calculate the percentage increases in intravascular P_mean_ (ΔP%) and flow (ΔFlow%) in each nidus vessel after SSA through each AF, with and without occlusion of all other AFs. We analyzed the observed shifts in hemodynamic compartments consequent to increasing AF injection pressures by mapping the hemodynamic changes onto a graphic representation of the nidus network using graded color scales to indicate hemodynamic values.

Next, we conducted a limited parameter sensitivity analysis of the model. We systematically altered two biophysical parameters in our model (vessel length and radius) to study the effects of a possible range of normal variations in AVM biophysical parameters on the behavior of this model and its fidelity to physiological reality. We randomly varied vessel length and radius 1,000 times throughout the entire network, sampling from a uniform distribution of values centered on the “typical” values listed in Supplementary Table 1, and within a 10% range of these values.

We also investigated if our findings pertaining to intranidal compartmentalization were dependent on the particular single nidus configuration we had studied, or if we could observe similar trends in compartmentalization in nidus networks of different geometries. Thus, we 100 times randomly varied the connections of 50% of the 97 AVM vessels. For each of these distinct morphologies, we simulated baseline state hemodynamics and SSA at each AF.

## Results

Hemodynamic simulations within the computational AVM model in its baseline state (i.e., when using the “typical” values for vessel length and radius stated in Supplementary Table 1) prior to simulated SSA revealed a total volumetric blood flow of 678 mL/min through the nidus akin to values obtained in large cerebral AVMs (Yamada et al., 1993), with markedly increased flow through the intranidal fistula. We observed high values of blood flow in DV2 primarily owing to the direct contribution from the intranidal fistula. Volumetric flow rate through individual plexiform vessels of the nidus ranged from <1 to 84.1 mL/min with a mean of 13.1 mL/min, while flow ranged from 34.3 to 145 mL/min with a mean of 98.9 mL/min through the fistulous vessels (Figure 1B). P_mean_ through the plexiform vessels of the nidus ranged from <1 to 103 mm Hg with a mean of 21.6 mm Hg, while P_mean_ ranged from <1 to 4.1 mm Hg with a mean of 2.9 mm Hg through the fistulous vessels (Figure 1C).

Sensitivity analysis of parameters showed that by varying the length and radius of AVM vessels by 10% above and below the “typical” simulation values, the volumetric blood flow through the AVM nidus ranged from 624 to 711 mL/min, and P_mean_ through the nidus ranged from 17.1 mm Hg to 26.2 mm Hg. Flow rate through the plexiform vessels of the nidus ranged from <1 to 95 mL/min, while flow ranged from 27.2 to 162 mL/min through the fistulous vessels. P_mean_ through the plexiform vessels of the nidus ranged from <1 to 124 mm Hg, while P_mean_ ranged from <1 to 6.1 mm Hg through the fistulous vessels. All these observed ranges were realistic and acceptable, indicating the robustness of the model to small variations in the vessel length and radius parameters used in constructing the model.

Superselective angiography through each AF resulted in the dissipation of intravascular P_mean_ to different extents within the nidus, demonstrating in effect the presence of distinct intranidal hemodynamic compartments depending on which AF was injected. We assessed the hemodynamic effects of each simulated SSA, with and without occlusion of all other AFs, by tabulating the consequent increases in P_mean_ value (and then calculating ΔP%) for each intranidal vessel. The average ΔP% values and sizes of compartments occurring after all SSAs, with and without occlusion of all other AFs, are seen in Supplementary Tables 2, 3, respectively.

**FIGURE 2:**
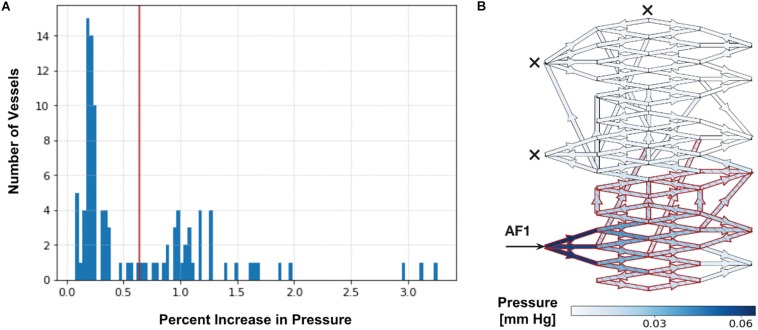
Determination of intranidal compartment size. **(A)** Tabulated results for P_mean_ above baseline (ΔP%) are first displayed graphically as a histogram. An example here illustrates a histogram of ΔP% values in intranidal vessels following a 20 mm Hg injection SSA into AF1 with simultaneous occlusion of all other AFs (as described in Supplementary Table 3). A bimodal distribution can be observed. The red line indicates the threshold value of ΔP% determined by Otsu’s method. Vessels with a ΔP% above this threshold (38% of nidus vessels) are considered part of a compartment. **(B)** Simulated distribution of P_mean_ throughout nidus vessels during a 20 mm Hg injection pressure SSA performed through AF1, with occlusion of all other AFs. Arrow indicates injection site and crosses indicate occluded AFs. Blue indicates nidus vessels experiencing a ΔP%. Superimposed red indicates nidus vessels forming part of the compartment served by the injected AF, as determined by Otsu’s method. Color scale shows range of ΔP% in mmHg. The ΔP in the plots is the change in pressure during injection over the non-injection state; it is a difference in pressure, and therefore has units of mm Hg. The ΔP is not a pressure drop across each vessel. The ΔP is useful to plot because it shows the change in pressure due to injection across the network, which depends on the flow and resistance throughout the network, structure of the network, and the site of injection.

Upon simulating each SSA (with and without occlusion of all other AFs), we observed a bimodal distribution in the range of ΔP% throughout vessels of the entire nidus, as seen in Figure 2. We applied Otsu’s clustering-based thresholding algorithm to this bimodal distribution (Otsu, 1979), which yielded a threshold value of percentage increase in ΔP (Figure 2). We considered the observed distribution or cluster of vessels having the higher ΔP% above the established threshold to represent those nidus vessels that were part of a functional or hemodynamic compartment supplied by the AF in which SSA was performed. The other distribution or cluster having the lower ΔP% below the established threshold represented vessels within the AVM nidus that were beyond this compartment. This allowed us to determine the percentage of the total number of nidus vessels involved in a particular hemodynamic compartment, i.e., the size of the compartment.

**FIGURE 3:**
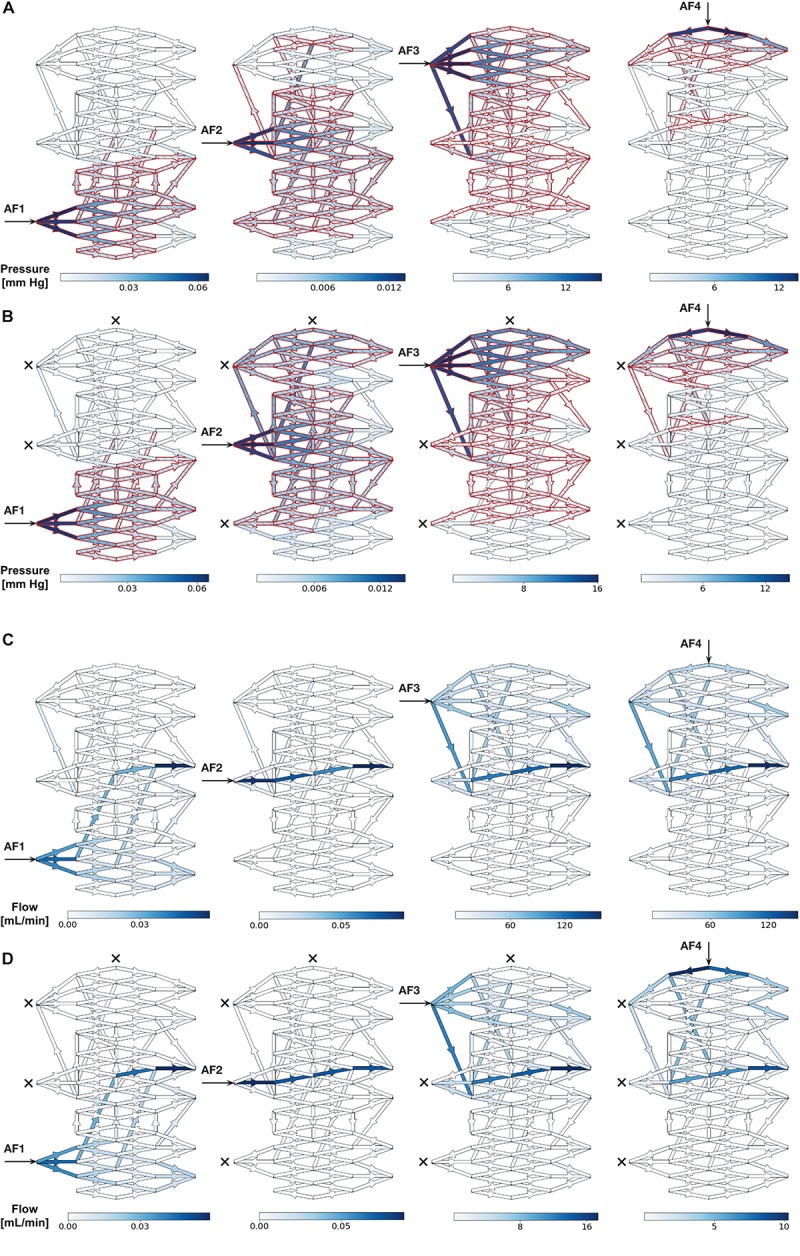
**(A,B)** Simulated distribution of P_mean_ throughout nidus vessels during SSA with a 20 mm Hg injection applied sequentially to each AF. **(A)** Each AF injection simulated without occlusion of any other AF. **(B)** Each AF injection simulated with occlusion of all other AFs. Arrows indicate injection site and crosses indicate occluded AFs. Blue indicates nidus vessels experiencing a change in P_mean_ above baseline (ΔP%). Superimposed red indicates nidus vessels forming part of the compartment served by the injected AF, as determined by Otsu’s method. Color scale shows range of pressures in mmHg. **(C,D)** Simulated distribution of flow throughout nidus vessels during SSA with a 20 mm Hg injection applied sequentially to each AF. **(C)** Each AF injection simulated without occlusion of any other AF. **(D)** Each AF injection simulated with occlusion of all other AFs. Arrows indicate injection site and crosses indicate occluded AFs. Blue indicates nidus vessels experiencing a change in flow above baseline (ΔFlow%). Color scale shows range of flows in mL/min.

Color maps of the changes in P_mean_ above baseline (ΔP%) superimposed onto the nidus network graphically revealed the changes in overall sizes of intranidal hemodynamic compartments with increasing injection pressures after SSA through each AF. As an example, the ΔP% values and sizes of intranidal compartments occurring after SSA, without and with occlusion of all other AFs, for injections at 20 mm Hg are illustrated graphically in Figures 3A,B. The corresponding change in ΔFlow% patterns within the nidus, also upon injections of 20 mm Hg, are shown in Figures 3C,D. Generally, the size of each nidus compartment was determined mainly by angioarchitectural variables (the number and type of intranidal vessels supplied by the AF through which a SSA was undertaken) rather than by the force of injections during SSA. The results of these SSA simulations are summarized in Table 1, which aids in interpreting the effects of the following variables on intranidal hemodynamics: injection pressures through AFs, AF location, AF type, and SSA with and without concurrent occlusion of other AFs.

**TABLE 1:** Summary of results from SSA simulations using the AVM model, and the effects on the sizes of intranidal compartments.

**Variables that are altered during SSA**	**Effects on the sizes of intranidal compartments**
• Injection pressures during SSA through each AF	Almost no change in compartment size in the range of simulated injection pressures
• The particular AF through which SSA is performed	Considerable effects on compartment size. The number of intranidal vessels each AF supplied was more important than the type of AF (minor or major, i.e., the input pressure into the nidus from each AF), or the SSA injection pressures, or type of intranidal vessels (plexiform or fistulous) in determining compartment size. See SSA through AF3 vs. AF2
• AF type (minor or major) through which SSA is performed	This was less important than the number of intranidal vessels each AF supplied in determining compartment size. See SSA through AF3 vs. AF2
• SSA into a plexiform vs. a fistulous portions of the nidus	Compartments were larger when SSA was performed into fistulous rather than plexiform vessels (e.g., AF2 injections > AF1, both are major AFs); when a larger number of intranidal vessels were supplied, regardless of them being plexiform or fistulous (AF3 > AF2); and when plexiform vessels were supplied by major feeder rather than a minor feeder (AF1 > AF4)
• SSA through each AF with concurrent occlusion of all other AFs	Compartments supplied by AF1, AF2, AF3, and AF4 showed increases in sizes of 0, 14, 4, and 30%, respectively, when compared to SSA with patent AVM feeders

As a departure from the single realization of the AVM network described above, our investigation of 100 versions of the AVM network, each with 50% of vessels randomly reconnected to different intranidal nodes, illustrated the robust and reproducible behavior of this particular model regardless of its angioarchitecture. In these 100 networks, blood flow through the AVM nidus ranged from 512 to 813 mL/min, and P_mean_ through the nidus ranged from 11.1 to 32.4 mm Hg. Flow rate through the plexiform vessels of the nidus ranged from <1 to 92 mL/min, while flow ranged from 22.5 to 181 mL/min through the fistulous vessels. P_mean_ through the plexiform vessels of the nidus ranged from <1 to 145 mm Hg, while P_mean_ ranged from <1 to 6.2 mm Hg through the fistulous vessels. All of these ranges were physiologically realistic. In 90% of the network geometries, during SSA we observed distributions in ΔP% that had a statistically significant bimodal nature (*p*-value of mean separation < 0.05).

As further validation, we repeated four times the same SSA simulation yielding the bimodal distribution of ΔP% depicted in Figure 2, but, instead, each time within a theoretical AVM nidus containing vessels randomly reconnected to different intranidal nodes (Supplementary Figure 1) than those depicted in Figure 2. Using Otsu’s method, these new simulations produced similar compartment sizes (extent of nidus affected varying from 38 to 46%) to that obtained in Figure 2 (i.e., 38%).

Taken together, this was clear evidence that our observation of intranidal compartmentalization was not strongly network dependent or owing to a particular choice of network geometry, but instead, was a general feature of our AVM network regardless of its internal architecture, thus reflecting the consistent clinical angiographic observation of intranidal compartments within large human AVMs.

## Discussion

The many advantages and limitations of theoretical AVM modeling based on electric network analysis have been discussed and commented upon in great detail previously by Hademenos et al. (1996), and are summarized in the Supplementary Material. The factors influencing the extent and hemodynamic features of intranidal compartments, as well as several important observations that can be made about AVM compartmentalization from simulations specifically using this model (summarized in Table 1) are also described in the Supplementary Material.

This model is of one single 3-D configuration, albeit an advance on prior modeling, and is constructed with implementation of average biophysical and hemodynamic parameters. In fact, values within a much wider range of AVM physiological and morphological parameters could have also been used, and would have likely yielded different outcomes. However, we did perform a limited parameter sensitivity analysis to confirm the authentic behavior of the AVM model when varying some of its biophysical inputs. A full sensitivity analysis of all AVM parameters (Hademenos and Massoud, 1996a) was beyond the scope of this initial presentation. Importantly, we also found that intranidal compartmentalization was maintained even if the network configurations within the nidus were varied widely, thus showing that the results observed in the single model we chose for in-depth analysis were not strongly network-dependent or owing to a particular choice of network geometry. Even though we were not able to test more datasets or architectures on our computational model owing to resource constraints, the significance of our results indicate generalizability to other datasets or architectures.

We do not wish to overstate the capability of this current model. Although many experimentally or clinically determined biophysical and hemodynamic parameters have been implemented in the model (with the aim of making it physiologically and clinically relevant), nonetheless the absence of concrete data to use for the nidus portion of the model, e.g., to accurately state length of nidus vessels, plays precisely into the “black box” concept that an AVM nidus represents. We used approximated values for nidus vessel length similar to that used by Hademenos et al. (1996) based on best “biophysical and anatomic reasoning.” This is a compromise in our modeling process, but clearly represents a limitation that, nonetheless, should be considered relative to the many other advantages that this computational model offers. We believe that the modeling process we present should act as a stimulus for further study of human AVM nidus biophysical, morphological, and morphometric characteristics that could then be implemented in future more clinically relevant AVM models to guide clinical decision making. The current work will also serve as a necessary basis and template for future more ambitious but clinically relevant AVM simulation projects.

For now, and despite the many limitations of current AVM modeling capabilities, we believe our study offers a theoretical AVM model that at least allows a qualitative appreciation of intranidal compartmentalization in a more rigorous manner than possible previously. The use of this model to help theoretically understand the behavior of AVM compartments is attractive in terms of its simplicity, intuitive familiarity (with some resemblance to clinically encountered AVMs), its implemented anatomic features, and the ease of its computer simulations. The visual display of our results in color-coded graphics superimposed on an image of an AVM nidus without a full need for mathematical comprehension of the computer simulations is an additional appealing feature. This model will be of benefit to clinical and research neuroscientists engaged in study and management of patients with AVMs.

## Conclusion

Applications of this novel 3-D AVM model to simulate SSA reveal that a combination of angioarchitectural features and hemodynamic equilibrium within the nidus (and not the injection pressures through AFs *per se*) are likely responsible for observed AVM hemodynamic compartmentalization (see also Supplementary Discussion Section 1.3). Importantly, and unlike what may be feasible when studying real AVMs, this biomathematical model uniquely allows theoretical manipulation of injection pressures, patency or occlusion of many AFs, and presence or absence of relevant angioarchitectural features in a manner that aids full theoretical interrogation of several biophysical and hemodynamic parameters simultaneously. Although our results using this single, specifically configured computational AVM model need to be appreciated in a qualitative manner mainly, we believe that the principles and methodology behind our study of intranidal hemodynamic compartmentalization indicate generalizability to other AVM model datasets or architectures. Our findings help to further understand and define some basic hemodynamic characteristics of brain AVMs, particularly within a region of an AVM (the nidus) that is seldom studied by other means, and the behavior of which is of paramount importance to various strategies that may be available for endovascular embolotherapy.

## Data Availability Statement

All datasets generated for this study are included in the manuscript/Supplementary Files.

## Author Contributions

TM conceived the study. All authors designed the experiments, interpreted and discussed the results, and reviewed, approved, and commented on the manuscript. TM and HD supervised the experiments. MJ and TM performed the experiments and wrote the manuscript.

## Conflict of Interest

The authors declare that the research was conducted in the absence of any commercial or financial relationships that could be construed as a potential conflict of interest.
