# Supplementary material for: Computational Network Modeling of Intranidal Hemodynamic Compartmentalization in a Theoretical Three-Dimensional Brain Arteriovenous Malformation
Source: Front Physiol. 2019 Sep 24;10:1250. doi: 10.3389/fphys.2019.01250 (PMC6769414; doi:10.3389/fphys.2019.01250)
Supplement: Supplementary file 1 [file Data_Sheet_1.PDF]

Computational Network Modeling of Intranidal Hemodynamic Compartmentalization in a Theoretical Three-Dimensional Brain Arteriovenous Malformation

SUPPLEMENTARY MATERIAL

1. Supplementary Discussion

Endovascular embolization can be used to treat small brain arteriovenous malformations (AVMs), or to substantially reduce the size of large AVMs through staged embolization prior to radiosurgery or microsurgical resection (Derdeyn et al., 2017). In either application, the AVM is assessed morphologically and hemodynamically with selective and superselective angiography (SSA). In SSA, contrast medium injection through microcatheters is performed within arterial feeders (AFs) as close as possible to the AVM nidus, revealing anatomical, functional, and hemodynamic information regarding portions of the AVM and its surroundings (Viñuela et al., 1984).

No current imaging or recording technique can provide precise hemodynamic information from within the nidus of a brain AVM. Owing to inaccessibility or danger of direct access to fragile intranidal vessels, such an assessment might be reduced to measuring blood flow and/or pressure outside a nidus using microcatheters placed in AFs and draining veins (DVs). Current imaging techniques are also limited in visually resolving individual nidus vessels when attempting to interrogate their flow noninvasively. Biomathematical models may provide convenient experimental tools that to some extent bridge this gap in knowledge by providing the means to notionally simulate and qualitatively appreciate the intranidal hemodynamic features of a theoretical AVM (Hademenos, et al., 1996). However, to date, reported AVM models have been simple representations, possessing small numbers of simulated intranidal vessels in rudimentary geometrical arrangements; these do not adequately represent the complex morphologies of large AVMs. Moreover, theoretical investigations of intranidal hemodynamics as they affect the behavior of intranidal functional compartments have not been attempted previously. In this study, we used a biomathematical AVM model based on the techniques of electrical network analysis and possessing a representative 3-D complex nidus architecture, to both simulate SSA at different injection pressures and to understand how this influences the consequent changes in intranidal hemodynamic compartments. There are no other techniques that could be used to interrogate hemodynamics deep within an AVM nidus, allowing at least a theoretical study of intranidal compartmentalization. A sound appreciation of AVM intranidal hemodynamics is necessary for basic understanding of these challenging cerebrovascular lesions, as well as being a prelude to future use of this model in theoretical simulations of endovascular embolotherapy and its hemodynamic influence on the nidus and surrounding brain.

The general merits and drawbacks of the prior simpler version of the model described by Hademenos et al. have been discussed previously, and these apply here similarly (reproduced here with permission) (Hademenos, et al., 1996).

The immense complexities of biological systems in reality prohibit true numerical analysis and quantitative modeling of inherent *in vivo* processes. Although true or sufficiently approximated hemodynamic and biophysical quantities were introduced and implemented in the simulations using this AVM model (to make this theoretical model as accurate and representative as possible), it has to be stressed that the results were intended to demonstrate only qualitative hemodynamic trends within

regions of an AVM nidus following SSA. Similarly, no direct quantitative extrapolation to a human AVM setting is possible with this model, but instead, only a theoretical appreciation can be gained for the ways by which contrast medium could spread within and traverse an AVM nidus.

Biomathematical modeling and simulations are useful as a means of providing the following: 1) a systematic and effective way of assembling existing knowledge about a system; 2) the identification of important parameters and determination of the overall system sensitivity to variation in each parameter; 3) the calculation of quantitative values of variables that are difficult or impossible to measure; 4) a method to rapidly, efficiently, and inexpensively test hypotheses; 5) the identification of specific elements or information gaps that must be further quantified, thus leading to the development of important experiments or quantitative measures; and 6) an effective model that can be used to predict the behavior of a real system. Each of these factors is relevant in the development of an AVM model.

The advantages presented by this model include the following: (1) a nidus with both plexiform and fistulous components that are interconnected and of a size typically found at histopathology are well represented anatomically, biophysically, and hemodynamically; (2) the AVM is fed by multiple AFs (two major feeders and two minor feeders, via the circle of Willis and the external carotid system) and drained by multiple veins; (3) the AVM model allows the investigation of hemodynamics under normal conditions and, therefore, may also allow the study of those changes occurring as a result of therapeutic interventions; (4) hemodynamic simulations are rapid and computationally efficient; (5) the AVM model can be easily amended to simulate the clinical presentation of any AVM by adjusting the number, size, length, location, and intravascular pressure of AFs and DVs; and (6) the AVM model not only reveals hemodynamic values within any feeding pedicle or nidus vessel but also provides information regarding intranidal compartments or areas of abnormal hemodynamics, which will be particularly important in future studies when using the model to investigate the risk of AVM nidus rupture.

Models are necessary to achieve reproducibility, which is an essential component of scientific experimentation. An overall drawback inherent to all theoretical modeling is the lack of biological traits and biovariability, a feature that may be found in naturally occurring or constructed *in vivo* models. Several specific limitations of this model are also evident, which nevertheless can be weighed against the many advantages it offers. These limitations include the polarity of nidus architecture, the pulsatility of blood flow, autoregulation, and the elementary design of the AVM network. Regarding the polarity of nidus architecture, in this model flow was assumed to proceed from the AFs (left) to the DVs (right) at opposite poles of the nidus, without recirculation or negative flow. In reality, AFs and DVs could arise into and from any location within the nidus. Regarding the pulsatility of blood flow, blood flow was described according to Poiseuille's law and, thus, simulates constant flow through rigid tubes. It is thought that pulsatility and the rate of increase of the pulsatile pressure waves in response to therapy could be a precursor to hemorrhagic episodes. Regarding autoregulation, controversy exists as to whether AVMs possess the capability to autoregulate blood flow through the nidus. According to Nornes and Grip (1980), “AVM vessels are viewed as fixed vascular conduits; they do not autoregulate in response to changes in arterial blood pressure and do not respond to chemical stimuli such as arterial CO_2. This suggests that increases in arterial blood pressure would be transmitted directly to AVM vessels.” However, in a study by Young et al. (1994), evidence was found to suggest that an AVM is capable of some as yet undefinable degree of autoregulation. Regarding the elementary design of the AVM network, although this AVM model based on network analysis represents a more intricate approach to replication of intracranial AVMs than is seen in previous models, the model is still rudimentary in its structural form and does not address the true complexity of a human AVM. Future more advanced computational modeling will be necessary on AVMs of greater complexity and realism.

Further studies will help elucidate the importance and influence of the above factors and will be considered in future simulations using our model.

1.1. Superselective angiography of AVMs

Angiography remains the gold standard for the structural and hemodynamic characterization of brain AVMs. SSA of brain AVMs was made possible by the efforts of several pioneers in neuroendovascular therapy, mainly, Serbinenko, Kerber, Pevsner, and Debrun (Viñuela et al., 1984). With development of supple microcatheters over the past three decades, it has been possible to superselectively catheterize second-, third-, and fourth-order branch vessels of the circle of Willis.

SSA allows the gathering of anatomical, dynamic, and functional information not available on less selective angiograms. Knowledge of this information is crucial for determining the position, rate, and strategy of embolic agent injection (Viñuela et al., 1984). Of significance within the context of this study is the particular information provided by SSA that may be characterized as both anatomic and dynamic; that is, the flow and spread through (and the volumetric extent of) that portion of the nidus supplied by the particular AF through which the SSA is performed. The resultant delineation of these various intranidal compartments using SSA is a reflection on the morphological characteristics of the AF, nidus, and DV as outlined by SSA and on the hemodynamic equilibrium present within the confines of the nidus.

In this study, each SSA was simulated by an increase in intravascular P_{mean} (as if during a hand injection) within each single corresponding AF supplying the AVM nidus. The intravascular pressure rise within the AF during SSA was assumed to vary between 10 mm Hg and 30 mm Hg. These elevations in pressure owing to the injections were deemed appropriately realistic values because of the small caliber of AFs, and these rises in pressure are typical of those measured in small vessels of experimental animals when using microcatheters (Saitoh et al. 1996). Furthermore, since the baseline P_{mean} in the major AFs of this model is 47 mm Hg, the assumed additional elevations due to SSA resulted in intravascular pressures that were still within limits for normal AF pressures recorded clinically (as reviewed previously by Hademenos, et al., 1996), i.e. they were not dangerously high. We therefore did not implement any simulations with injection pressures higher than 30 mm Hg lest these be considered unrealistic and contributory to potential AF or nidus rupture. The above described strategies of performing SSA through AFs were used in this study to investigate the consequent hemodynamic effects downstream within the nidus and the nature of intranidal hemodynamic compartments.

1.2. Intranidal hemodynamic compartmentalization

The concept of an AVM nidus was advanced initially by Doppman (1971) who stated "the nidus is the point toward which multiple feeding arteries converge and from which the enlarged veins drain. It is in essence the fundamental lesion or the basic vascular pathology". Using modern imaging techniques, it is a routine task to acquire information regarding the location, size, shape, and anatomical relationships of the nidus of a brain AVM. However, comparatively little attention to date has been paid, or even been possible, to the scientific analysis of intranidal hemodynamics, and specifically to the issue of intranidal compartmentalization—both are difficult to investigate by means other than by theoretical models.

According to the literature, the nidus of a large AVM may be compartmentalized either structurally or hemodynamically. Structural compartmentalization (also called "sectorization" by Pertuiset et al. (1982). assumes that groups of nidus vessels are morphologically independent from other vessels within the nidus in the form of separate sectors or compartments. This would imply that

no recirculation or redistribution of hemodynamic forces occurs between compartments upon any perturbation of flow within the AVM nidus. True structural compartmentalization is relatively uncommon in AVMs, observed in 3 out of 57 patients in one study (Pertuiset et al., 1982). Hemodynamic compartmentalization (described by many authors) (Massoud and Hademenos, 1999), on the other hand, involves groups of nidus vessels that are connected anatomically but where blood mixing does not occur during transit from the AF to the DV owing to a hemodynamic equilibrium between functional compartments. Each hemodynamic compartment has a separate AF and is drained by one or more DVs. If an AF divides to subserve two different portions of the nidus, these are considered as two separate arterial pedicles supplying two distinct hemodynamic compartments. Sometimes DVs from separate compartments are joined to form one major DV, and hence, the number of DVs of a large AVM is usually less than the number of the AFs (Patronas, 1980). An AVM nidus of less than 3 cm in diameter usually has only one compartment, a reflection on the one or two AFs that usually supply AVMs of this size; but as the nidus increases in size, so do the number of hemodynamic compartments.

Within a nidus, the various compartments are in hemodynamic balance. Thus, if one AF is occluded and the nidus it supplies remains patent, the portion of the nidus fed previously by the occluded AF will continue to receive blood from adjacent regions of the nidus, as demonstrated in the past by ApSimon and Khangure (1986), and Abe et al. (1989). These adjacent regions represent compartments that expand in size to overtake that portion of the nidus depleted of blood. For this reason, Yaşargil (1987a) had stated previously that *any meaningful in vivo analysis of AVM hemodynamic compartments (their size, extent of normal overlap, and potential for expansion into adjacent compartments) would require that all other AFs to a nidus be occluded temporarily while one of the multiple AFs entering the nidus is being tested with contrast material*; a difficult or even impossible task in practice *in vivo* when dealing with a large AVM supplied by numerous feeders. Notwithstanding, the hemodynamic behavior of blood or contrast medium within an AVM nidus has important implications regarding the various strategies that can be adopted for the superselective delivery of embolic agents to occlude a nidus.

1.3. Factors influencing intranidal compartmentalization in our model simulations

With the background knowledge that shunting flow through an AVM is most likely passive and pressure-dependent, i.e. lacking autoregulation (Nornes and Grip, 1980), several important observations (summarized in the published article, Table 1) can be made about AVM compartmentalization from simulations specifically using this model. First, the spatial location of the collection of intranidal vessels subject to ΔP% above a threshold (i.e. the extent of the compartment) is well defined and is adjacent to the AF through which the injection is performed. This collection was the cluster of nidus vessels having a higher ΔP% in each described bimodal distribution. The demonstration of these well-delineated intranidal hemodynamic compartments (i.e. the vessels affected by the increase in pressure were not scattered randomly throughout the nidus) that do not encroach on adjacent compartments supplied by other AFs, is reminiscent of the clinical SSA visualization by Kakizawa et al. (2002) of distinct functional AVM compartments, seen on post-processed, color-coded patient angiograms, that are in hemodynamic equilibrium within the nidus, and attests further to the realistic behavior of the AVM model we present herein.

Second, in the specific configuration of this AVM model that we studied in depth, an increase in the injection pressures in any of the AFs from 10 mm Hg to 20 mm Hg or 30 mm Hg resulted in intranidal compartment size increases of <2%. Thus, only a minimal increase in the size of a nidus compartment occurs with considerable increases in injection pressures through its AF. Intranidal

angioarchitectural features, therefore, appear from a theoretical standpoint to be more important factors than the force of injection at SSA (in the safe ranges encountered clinically) in determining the spread of contrast medium through the nidus. As described in published article Table 1, larger intranidal compartments were observed: (1) when an AF divides into and supplies a larger number of intranidal vessels, as seen upon SSA through AF3, compared to that through AF2; (2) when SSA is performed into a fistulous rather than a plexiform portion of the nidus, e.g. SSA through AF2 compared to AF1; and (3) when plexiform vessels were supplied by major AFs at higher baseline pressure, than by minor AFs at lower baseline pressure, e.g. SSA through AF1 compared to AF4.

With regard to simulations involving the intranidal fistula, we observed larger compartments when SSA was performed through AF2 compared with AF1. However, in the particular configuration of this model, we found that SSA into AF3 produced a larger compartment than an injection through AF2. Therefore, the number of intranidal vessels supplied by each AF was more important than the type of intranidal vessel (plexiform or fistulous) in determining compartment size. This may seem counterintuitive at first; after all, the intranidal fistula might be thought of as a wider conduit that could allow a greater extent of dissemination of the injection pressure within the nidus to most of its volume on account of it representing a path of least resistance within the confines of the nidus. However, we believe that in fact the behavior of this fistula within this AVM model is indeed realistic because the likely presence of a strong sump effect caused by DV2 connected to the intranidal fistula (with its low pressure and high flow) likely outweighs and lessens the possible spread of an injection pressure wave into adjacent plexiform vessels to allow the formation of a large intranidal compartment. The injection pressure wave originating in AF2 likely travels through the fistula, influenced primarily by this venous sump effect. Reassuringly, the intranidal flow simulations through all AFs reveal distinct channeling of flow toward the fistula, and prominent rises above baseline flow values within the intranidal fistula itself as it approaches DV2 (published article Figure 3), somewhat reminiscent of the clinical scenario of preferential flow through an intranidal fistula when SSA is performed in AVMs of patients. Of note, the configuration of the intranidal fistula in our particular model is uncommon in clinical practice (and is therefore a relative drawback of our model). Some 40% of fistulas in AVMs proceed directly to their DVs without anastomosing with adjacent plexiform vessels, whether the fistula is intra- or extranidal (Yuki et al., 2010). We will study the effects of these different fistula morphologies in future more detailed analyses.

When SSA was simulated through each AF with concurrent occlusion of all other AFs (See Tables 2 and 3 below), the patterns obtained were a reflection on the particular 3-D geometry of this nidus network we have, but we do observe that for all AFs, their occlusion does increase the extent of nidus filling. We had opted to provide this information in the tables as well as Figure 3 of the main paper because it is hard to see this increase at first glance in the figure alone, since it would require counting the vessels. Nonetheless, two general observations can be made. First, all compartments maintained their general integrity (remaining in close proximity to each supplying AF) without the intranidal vessels affected by $\Delta P\%$ scattering randomly throughout the nidus. Second, there was usually an increase in extent of nidus filling, i.e. an increase in the size of each compartment, varying from 0% to 30%. Of note, AF4 (a simulated transdural external carotid AF) supplied the smallest intranidal compartment at baseline, but this compartment benefited the most by pressure redistribution and it expanded the most after SSA during occlusion of the other AFs. The opposite can be said for AF3. However, the full reasons why SSA through AF1 produced no change in compartment size remains unclear. A detailed multivariate analysis of all possible biophysical and hemodynamic factors determining the size of each compartment was beyond the scope of this study, and will be the subject of future more detailed investigations.

One would expect all intranidal compartments to be in hemodynamic equilibrium (thus filling 100% of the nidus, without overlap of compartments) when the AVM is in its baseline state (at

normotension in all AFs). That would be the case without the addition of any superselective angiographic increases in intravascular pressures through the AFs. However, we have simulated SSA injections through the four AFs, in turn, at pressures varying from 10-30 mmHg. These simulations showed that the compartment sizes (extent of nidus affected, shown in Supplementary Tables 2 and 3), when combined, do add up to >100%, even with a 10 mmHg SSA injection. Although the 10-30 mmHg range for SSA injections that we used is derived from in vivo experimental data (Saitoh et al. 1996), we believe that it may be a function of the particular 3-D geometry of this nidus network that even a 10 mmHg SSA injection through any AF results in a large outlined compartment, such that a similar 10 mmHg injection through different AFs would result in apparent overlap of compartments. We believe this needs to be investigated further in a future more rigorous study that uses this model plus many other ones of different configurations, to simulate (1) the size of the compartments at decremental SSAs of 10 mmHg down to 1 mmHg through each AF in turn (to see if this apparent overlap diminishes down close to values that indicate no overlap when such SSAs entail increases in AF pressures closer to 1 mmHg); and (2) the size of the compartments at incremental SSAs of 1-30 mmHg simultaneously through all four AFs (to also see how this affects the apparent overlap of compartments, or, as might be expected, it would restore the full equilibrium between compartments to result in no overlap).

Taken together, the experimental manipulations made possible by this model, allowing concurrent occlusion of all AFs other than the one through which SSA is performed, now provides a detailed theoretical validation of the concept of hemodynamic compartmentalization in the manner first proposed by Yaşargil (1987) over three decades ago, but which has not been possible to carry out until now.

1.4. References (for Supplementary Discussion)

1. Derdeyn CP, Zipfel GJ, Albuquerque FC. Management of brain arteriovenous malformations: A scientific statement for healthcare professionals from the American Heart Association/American Stroke Association. *Stroke* 2017, 48, e200-e224.

2. Viñuela F, Fox AJ, Debrun G, Pelz D. Preembolization superselective angiography: role in the treatment of brain arteriovenous malformations with isobutyl-2 cyanoacrylate. *AJNR Am J Neuroradiol*. 1984, 5, 765-769.

3. Hademenos GJ, Massoud TF, Viñuela F. A biomathematical model of intracranial arteriovenous malformations based on electrical network analysis: theory and hemodynamics. *Neurosurgery* 1996, 38, 1005-1014; discussion 1014-1015.

4. Nornes H, Grip A. Hemodynamic aspects of cerebral arteriovenous malformations. *J Neurosurg*. 1980, 53, 456-64.

5. Young WL, Pile-Spellman J, Prohovnik I, Kader A, Stein BM, the Columbia University AVM Study Project. Evidence for adaptive autoregulatory displacement in hypotensive cortical territories adjacent to arteriovenous malformations. *Neurosurgery* 34, 601-611, 1994.

6. Saitoh H, Hayakawa K, Nishimura K, et al. Intracarotid blood pressure changes during contrast medium injection. *AJNR Am J Neuroradiol.* 1996, 17, 51-54.

7. Doppman JL. The nidus concept of spinal cord arteriovenous malformations. A surgical recommendation based upon angiographic observations. *Br J Radiol*. 1971, 44, 758-763.

8. Pertuiset B, Ancri D, Clergue F. Preoperative evaluation of hemodynamic factors in cerebral arteriovenous malformations for selection of a radical surgery tactic with special reference to vascular autoregulation disorders. *Neurol Res*. 1982, 4, 209-233.

9. Massoud TF, Hademenos GJ. Transvenous retrograde nidus sclerotherapy under controlled hypotension (TRENSH): A newly proposed treatment for brain arteriovenous malformations—concepts and rationale. *Neurosurgery* 1999, 45, 351-63; discussion 363-5.

10. Patronas NJ, Marx WJ, Duda EE, Mullan JJ. Microvascular embolization of arteriovenous malformations: Predicting success by cerebral angiography. *AJNR Am J Neuroradiol.* 1980, 1, 459-462.

11. ApSimon HT, Khangure MS. Improved technique of bucrylate embolisation in brain arteriovenous malformation. The use of additional temporary balloon occlusion. *Acta Radiol* Suppl. 1986, 369, 618-20.

12. Abe H, Koike T, Minakawa T, Tanaka R. Liquid embolization for arteriovenous malformation with temporary balloon occlusion of another feeders. *Rinsho Hoshasen*. 1989, 34, 739-42.

13. Yasargil MG. *Angiographic Investigation*. In: Yasargil MG, ed. *Microneurosurgery IIIA*. Stuttgart: Georg Thieme Verlag; 1987, 269-283.

14. Kakizawa Y, Nagashima H, Oya F. Compartments in arteriovenous malformation nidi demonstrated with rotational three-dimensional digital subtraction angiography by using selective microcatheterization. Report of three cases. *J Neurosurg* 2002, 96, 770-774.

15. Yuki I, Kim RH, Duckwiler G, et al. Treatment of brain arteriovenous malformations with high-flow arteriovenous fistulas: risk and complications associated with endovascular embolization in multimodality treatment. *J Neurosurg* 2010, 113, 715-722.

2. Supplementary Figure

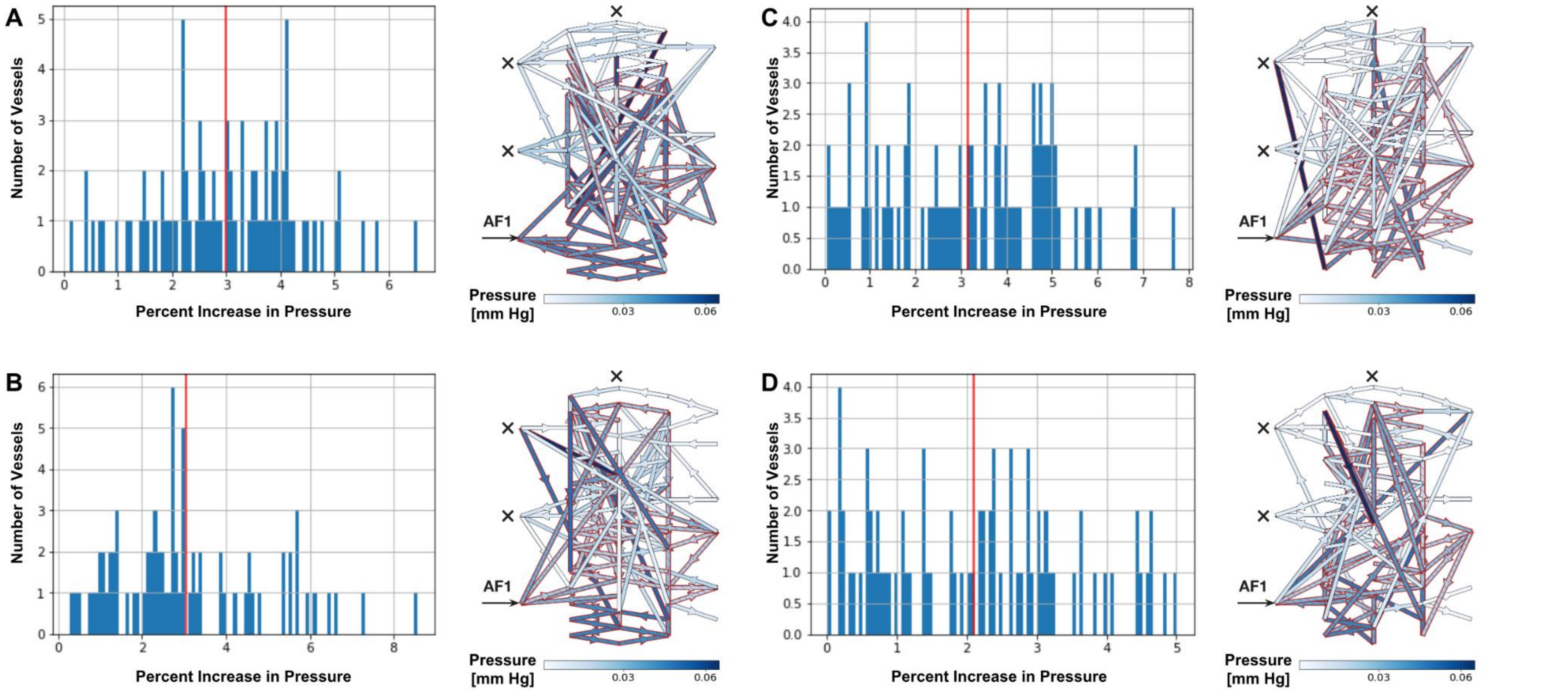

Supplementary Figure 1

Determination of intranidal compartment size. **A.** Tabulated results for P_{mean} above baseline (ΔP%) are first displayed graphically as a histogram. Four examples of different AVMs (A to D) here illustrate histograms of ΔP% values in intranidal vessels following a 20 mm Hg injection SSA into AF1 with simultaneous occlusion of all other AFs (as described in Supplementary Table 3). A bimodal distribution can be observed. The red line indicates the threshold value of ΔP% determined by Otsu's method. Vessels with a ΔP% above this threshold (39%, 46%, 42%, and 38% for nidus A to D, respectively) are considered part of a compartment within each nidus. Simulated distribution of P_{mean}

values throughout nidus vessels during a 20 mm Hg injection pressure SSA performed through AF1, with occlusion of all other AFs, are shown to the right of each histogram. Arrow indicates injection site and crosses indicate occluded AFs. Blue indicates nidus vessels experiencing a ΔP%. Superimposed red indicates nidus vessels forming part of the compartment served by the injected AF, as determined by Otsu's method. Color scale shows range of ΔP% in mmHg.

3. Supplementary Tables

3.1. Supplementary Table 1

Anatomic and biophysical parameters for the blood vessels represented in the biomathematical AVM model, as per Hademenos et al. (1996) in the published article.

**Vessel**	**R** (cm)	**L** (cm)	$\mathbf{R_v}$ (dyne s/cm^5)
Cardiovasculature			
N32 – E_{SP} (superior vena cava)	0.750	10.0	3.2
E_{SP} – N1 (aortic arch)	1.000	10.0	1.0
N1 – N2 (subclavian artery)	0.350	10.0	67.9
Head and neck vasculature			
Neck and extracranial circulation	0.350	10.0	67.9
N1 – N4 (common carotid artery)	0.200	10.0	637.5
N4 – N5 (external carotid artery)	CP bed	CP bed	1000000.0
N5 – N9	CP bed	CP bed	1000000.0
N9 – N10	0.125	10.0	4177.9
N31 – N32 (jugular veins)	0.400	20.0	79.7
Intracranial circulation			
N4 – N6 (internal carotid artery)	0.250	20.0	522.0
N2 – N3 (vertebral artery)	0.150	25.0	5037.0
N6 – N7	0.100	10.0	10200.0
N7 – N8	CP bed	CP bed	1000000.0
N8 – N11	0.125	10.0	4177.9
N11 – N31 (dural venous sinuses)	0.250	10.0	261.0
AVM vasculature			

Major arterial feeders			
AF1 (posterior cerebral artery)	0.125	5.2	2210.0
AF2 (middle cerebral artery)	0.150	3.7	745.5
Minor arterial feeders			
AF3 (anterior cerebral artery)	0.025	3.7	15725000.0
AF4 (transdural feeding artery)	0.0125	3.0	12750000.0
Nidus vessels			
Plexiform	0.050	5.0	81600.0
Fistulous	0.100	4.0	4080.0
Draining veins			
DV1	0.250	5.0	130.5
DV2	0.250	5.0	130.5
DV3	0.250	5.0	130.5

*** R, vessel radius; L, vessel length; R_v, vascular resistance; N, node; E_{SP}, systemic arterial blood pressure; AF, arterial feeder; DV, draining vein; CP capillary.**

3.2. Supplementary Table 2

Superselective angiography: Injections through each arterial feeder in turn while all other three arterial feeders were patent

Arterial Feeder	Injection Pressure (mm Hg)	Extent of Nidus Affected (%)	ΔP (%)
AF1	10	38	1.4
AF1	20	38	2.8
AF1	30	38	4.2
AF2	10	65	0.2
AF2	20	66	0.3
AF2	30	66	0.5
AF3	10	76	82
AF3	20	76	164
AF3	30	76	246
AF4	10	20	15
AF4	20	20	29
AF4	30	20	44

3.3. Supplementary Table 3

Superselective angiography: Injections through each arterial feeder in turn, when simultaneously occluding the other three arterial feeders

Arterial Feeder	Injection Pressure (mm Hg)	Extent of Nidus Affected (%)	ΔP (%)
AF1	10	38	0.04
AF1	20	38	0.08
AF1	30	39	0.11
AF2	10	76	0.02
AF2	20	75	0.03
AF2	30	75	0.05
AF3	10	79	31.7
AF3	20	79	63.3
AF3	30	79	95.0
AF4	10	26	23.9
AF4	20	26	47.8
AF4	30	26	71.6
